# 2018 JAPAN Critical Limb Ischemia Database (JCLIMB) Annual Report

**DOI:** 10.3400/avd.ar.21-00047

**Published:** 2021-06-25

**Authors:** The Japanese Society for Vascular Surgery JCLIMB Committee, NCD JCLIMB Analytical Team

**Keywords:** arterial occlusive disease, leg ischemia, peripheral arterial disease, critical limb ischemia, annual report

## Abstract

Since 2013, the Japanese Society for Vascular Surgery has started the project of nationwide registration and tracking database for patients with critical limb ischemia (CLI) who are treated by vascular surgeons. The purpose of this project is to clarify the current status of the medical practice for patients with CLI to contribute to the improvement of the quality of medical care. This database, called JAPAN CLI Database (JCLIMB), is created on the National Clinical Database and collects data of patients’ background, therapeutic measures, early results, and long-term prognosis as long as 5 years after the initial treatment. The limbs managed conservatively are also registered in the JCLIMB, together with those treated by surgery and/or endovascular treatment. In 2018, 1,145 CLI limbs (male 758 limbs, 66%) were registered by 90 facilities. Arteriosclerosis obliterans has accounted for 97% of the pathogenesis of these limbs. In this manuscript, the background data, ischemic status, treatment, and the early prognosis (within 1 month) of the registered limbs are reported. (This is a translation of Jpn J Vasc Surg 2020; 29: 365–393.)

## 1. Introduction

Recently, an increasing number of patients with critical limb ischemia (CLI) are undergoing medical care at clinical practice sites. Improving the outcome of treatment for these patients is an important and urgent issue. Since 2013, the Japanese Society for Vascular Surgery (JSVS) has initiated the project of a nationwide CLI registration and tracking database to obtain CLI epidemiological data that can be shared among the medical staff. The background of CLI limbs, contents of treatment, early outcome, and long-term outcome until 5 years after surgery, including non-surgical limbs, are registered in this database. The database was named JAPAN CLI Database (JCLIMB) and established on the National Clinical Database (NCD). The JCLIMB project’s primary objective is to clarify the current status of CLI treatment performed by vascular surgeons in Japan and inform physicians at practice sites, thus improving the quality of medical care. The initial registration data, and their tracking data 1 month after registration in 2013–2017, have already been published.^1–5)^ This article reports the basic data registered in 2018.

## 2. JCLIMB

Registration details, including the definition of CLI, have already been described in the 2013 annual report.^1)^ CLI to be registered was defined according to the TASC II classification^6)^: chronic ischemic rest pain, ulcers, or gangrene attributable to objectively proven arterial occlusive disease. CLI diagnosis should be confirmed by ankle pressure (AP) below 50 mmHg or by toe pressure (TP) below 30 mmHg in the limbs with rest pain and done by AP below 70 mmHg or by TP below 50 mmHg in the limbs with ulcer or gangrene.

The same limb can be registered in the JCLIMB only once within a 5-year tracking period. When the registered limb is treated at different times or at different institutions, such data should be added only to the tracking items of each limb in the JCLIMB, avoiding registration overlap. However, details of the procedure are registered each time in the NCD apart from the registration in the JCLIMB. On the other hand, the patient with bilateral CLI can be registered twice for each limb. Based on the NCD regulations, fixing the JCLIMB data are done as follows:

Initial registration data: early April in the following year, tracking data early after treatment (1 month)/6 months after treatment: end of December in the following year, tracking data 1 year after treatment: end of December after 2 years.

Tracking data 2 years after treatment: end of December after 3 years

Tracking data 3 years after treatment: end of December after 4 years

Tracking data 4 years after treatment: end of December after 5 years

Tracking data 5 years after treatment: end of December after 6 years.

As a general rule, the timing of tracking data registration is accepted within a ±2-month range until 12 months after treatment and within a ±3-month range thereafter. Although the day for tracking data fixing is specified, it is made flexible because, in some limbs, follow-up data might be revealed later.

It is very difficult to require facilities participating in the NCD to register CLI data since a great number of registration items in the JCLIMB would put too much burden on them. Thus, facilities wishing to participate were recruited. In total, 90 facilities, which registered CLI limbs in 2018 at the time of compiling in September 2020, are listed in the appendix.

Since the JCLIMB is positioned as a registry study on the NCD, patient consent to participate in the study and the ethical review of the study at the time of participation in the NCD were adopted.

## 3. Comments on the Aggregated Data in 2018

The initial registration data in 2018 were fixed in early April 2019, and the tracking data early after treatment (1 month) were fixed on December 31, 2019. At that time, 1,145 limbs, those of 758 males (66%) and 387 females (34%), were registered in 90 facilities. All data and extracted data on arteriosclerosis obliterans (ASO) were collected according to the registered items. Since ASO accounted for 97% of all limbs, the overall and ASO data showed similar tendencies. In the comments, ASO data were presented in parentheses. In addition, because the Society for Vascular Surgery’s (SVS) WIfI classification was reported in 2014 (**Tables 1-1-1** to **1-1-3**),^7)^ the JCLIMB made several changes and additions to the registered items, making the WIfI classification possible since 2015 (**Tables 1-2-1** to **1-2-3**). The total figure was not always consistent, mostly due to missing values, and an explanation for each inconsistency was added.

**Table:** Table 1-1 SVS WIfI classification original^5）^ Table 1-1-1 Wound

Grade	Ulcer	Gangrene
0	No ulcer No gangrene	No gangrene
	Clinical description: ischemic rest pain (requires typical symptoms. ischemia grade 3); no wound.	
1	Small, shallow ulcer(s) on distal leg or foot; no exposed bone, unless limited to distal phalanx	No gangrene
	Clinical description: minor tissue loss. Salvageable with simple digital amputation (1 or 2 digits) or skin coverage.	
2	Deeper ulcer with exposed bone, joint or tendon; generally not involving the heel; Gangrenous changes limited to digits shallow heel ulcer, without calcaneal involvement	Gangrenous changes limited to digits
	Clinical description: major tissue loss salvageable with multiple (3) digital amputations or standard TMA±skin coverage.	
3	Extensive, deep ulcer involving forefoot and/or midfoot; deep, full thickness heel ulcer±calcaneal involvement	Extensive gangrene involving forefoot and/or midfoot; full thickness heel necrosis 6 calcaneal involvement
	Clinical description: extensive tissue loss salvageable only with a complex foot reconstruction or nontraditional TMA (Chopart or Lisfranc); flap coverage or complex wound management needed for large soft tissue defect	

TMA: transmetatarsal amputation

**Table:** Table 1-1-2 Ischemia

Grade	ABI	AP (mmHg)	TP, TcPO_2_ (mmHg)
0	≥0.80	>100	≥60
1	0.60–0.79	70–100	40–59
2	0.40–0.59	50–70	30–39
3	≤0.39	<50	<30

ABI: ankle brachial (pressure) index, PVR: pulse volume recording, SPP: skin perfusion pressure, TP: toe pressure, TcPO_2_: transcutaneous oximetry Patients with diabetes should have TP measurements. If arterial calcification precludes reliable ABI or TP measurements, ischemia should be documented by TcPO_2_, SPP, PVR. If TP and ABI measurements result or in different grades, TP will be the primary determinant of ischemia grade. Flat or minimally pulsatile forefoot PVR=grade 3.

**Table:** Table 1-1-3 Foot infection

Grade	Clinical manifestation of infection	IDSA/PEDIS infection severity*
0	No symptoms or signs of infection	Uninfected
1	Infection present, as defined by the presence of at least 2 of the following items: Mild	Mild
	·Local swelling or induration	
	·Erythema >0.5 to 2 cm around the ulcer	
	·Local tenderness or pain	
	·Local warmth	
	·Purulent discharge (thick, opaque to white, or sanguineous secretion)	
	Local infection involving only the skin and the subcutaneous tissue (without involvement of deeper tissues and without systemic signs as described below).	
	Exclude other causes of an inflammatory response of the skin (e.g., trauma, gout, acute Charcot neuro-osteoarthropathy, fracture, thrombosis, venous stasis)	
2	Local infection (as described above) with erythema >2 cm, or involving structures deeper than skin and subcutaneous tissues (e.g., abscess, osteomyelitis, septic arthritis, fasciitis), and no systemic inflammatory response signs (as described below)	Moderate
3	Local infection (as described above) with the signs of SIRS, as manifested by two or more of the following:	Severe
	·Temperature >38°C or <36°C	
	·Heart rate >90 beats/min	
	·Respiratory rate >20 breaths/min or PaCO_2_ <32 mmHg	
	·White blood cell count >12,000 or <4,000 cu/mm or 10% immature (band) forms	

*SVS adaptation of Infectious Diseases Society of America (IDSA) and International Working Group on the Diabetic Foot (IWGDF) perfusion, extent/size, PACO_2_: Partial pressure of arterial carbon dioxide, SIRS: systemic inflammatory response syndrome. An Ischemia may complicate and increase the severity of any infection. Systemic infection may sometimes manifest with other clinical findings, such as hypo-tension, confusion, vomiting, or evidence of metabolic disturbances, such as acidosis, severe hyperglycemia, new-onset azotemia.

**Table:** Table 1-2 SVS WIfI classification: Correlation of WIfI and items in JCLIMB Table 1-2-1 Wound

Grade	Rutherford classification	Ulcer		Sites of gangrene
		Depth of ulcer (University of Texas classification: grade)	Sites of ulcer	
0	Class 4		No ulcer	No gangrene
1	Class 5, 6	I	Any portion	No gangrene
		II, III	Limited to digits	
2	Class 5, 6	I	Heel	Limited to digits
		II, III	Foot: distal metatarsal excluding heel	
3	Class 5, 6	II, III	Foot: proximal metatarsal, heel, ankle, lower leg	Extensive proximal to fore foot

**Table:** Table 1-2-2 Ischemia

Grade	SPP: (mmHg; calculating from the formula*)
0	>55
1	42–55
2	35–41
3	<35

*SPP=0.6853×TP+14.48 SPP: skin perfusion pressure, TP: toe pressure

**Table:** Table 1-2-3 Foot infection

Grade	Local infection; foot	Systemic infection (SIRS)
0	(−)	(−)
1	(+)	(−)
	Involving only the skin and the subcutaneous tissue (Erythema around the ulcer; 0.5–2 cm)	
2	(+)	(−)
	Involving only the skin and the subcutaneous tissue (Erythema around the ulcer; >2 cm), or involving structures deeper than skin and subcutaneous tissues (e.g., abscess, osteomyelitis, septic arthritis, fasciitis)	
3	(+)	(+)

### (1) Pretreatment patients’ background

Pretreatment patients’ background is shown in **Tables 2-1** to **2-6**. Good blood pressure control was defined as below 140/90 mmHg, without diabetes and renal failure, or below 130/80 mmHg with these diseases. Diabetes control was considered good when hemoglobin A1c was below 7.0% (National Glycohemoglobin Standardization Program value). Dyslipidemia control was considered good when low-density lipoprotein was below 100 and 80 mg/dL in the absence and presence of other arteriosclerotic diseases, respectively. The presence of heart failure was judged clinically. The patient was regarded as having heart failure based on a history of admission due to heart failure, clinical symptoms of heart failure, a diagnosis of heart failure was confirmed by echocardiography, or reduced cardiac function on echocardiography even with no clinical heart failure symptoms. Renal dysfunction was graded following the new chronic kidney disease severity classification of the “Clinical Practice Guidebook for Diagnosis and Treatment of Chronic Kidney Disease 2012”^8)^: renal dysfunction was absent when the estimated glomerular filtration rate (eGFR) (mL/min/1.73 m^2^) was 60 or higher, and it was graded as G3a, G3b, G4, and G5 when eGFR was 45–59, 30–44, 15–29, and below 15, respectively. An eGFR below 15 in hemodialysis patients was graded as G5D.

**Table:** Table 2 Patients’ background Table 2-1 Patients’ background 1

a. Total																		
	n	Sex		Laterality		BMI (Median)	Pathogenesis				Age at registration							
		Male	Female	Right	Left		ASO	TAO	Vasculitis	Others	ASO		TAO		Vasculitis		Others	
											Mean (±SD)		Mean (±SD)		Mean (±SD)		Mean (±SD)	
Rutherford 4	199	134	65	98	101	21.0	189	3	3	4	75.2	(8.6)	36.7	(10.7)	63.0	(5.0)	68.3	(8.2)
Rutherford 5	761	499	262	388	373	21.2	738	1	11	11	74.3	(9.9)	43.0	—	68.4	(11.0)	59.0	(15.2)
Rutherford 6	185	125	60	90	95	20.3	178	0	5	2	72.4	(10.9)	—	—	68.8	(12.5)	59.0	(12.7)
Total	1145	758	387	576	569	21.1	1105	4	19	17	74.2	(9.9)	38.3	(9.3)	67.6	(10.4)	61.2	(13.5)
b. ASO																		
	n	Sex		Laterality		BMI (Median)	Age at registration											
		Male	Female	Right	Left		Mean (±SD)											
Rutherford 4	189	125	64	91	98	21.0	75.2	(8.6)										
Rutherford 5	738	488	250	374	364	21.2	74.3	(9.9)										
Rutherford 6	178	120	58	87	91	20.3	72.4	(10.9)										
Total	1105	733	372	552	553	21.1	74.2	(9.9)										

Vasculitis: Takayasu’s arteritis, collagen disease, Behcet disease, FMD etc., excluding TAO Others: others (including debranch bypasses for TEVAR or EVAR) ASO: arteriosclerosis obliterans, TAO: thromboangiitis obliterans, FMD: fibromuscular dysplasia, BMI: body mass index, TEVAR: thoracic endovascular aortic/aneurysm repair, EVAR: endovascular aortic/aneurysm repair

**Table:** Table 2-2 Patients’ background 2

a. Total															
	Diabetes			Diabetes therapy			Hypertension			Dyslipidemia			Smoking		
	(−)	(+)		Diet therapy	Medication	Insulin therapy	(−)	(+)		(−)	(+)		(−)	(+)	
		Management						Management			Management			Ex-smoker	Current smoker
		Good	Poor					Good	Poor		Good	Poor			
Rutherford 4	100	76	23	12	50	37	57	127	15	114	76	9	84	90	25
Rutherford 5	250	390	121	72	255	184	171	494	96	443	278	40	340	325	96
Rutherford 6	56	89	40	18	61	50	47	112	26	117	64	4	68	87	30
Total	406	555	184	102	366	271	275	733	137	674	418	53	492	502	151
b. ASO															
	Diabetes			Diabetes therapy			Hypertension			Dyslipidemia			Smoking		
	(−)	(+)		Diet therapy	Medication	Insulin therapy	(−)	(+)		(−)	(+)		(−)	(+)	
		Management						Management			Management			Ex-smoker	Current smoker
		Good	Poor					Good	Poor		Good	Poor			
Rutherford 4	95	72	22	9	48	37	49	125	15	106	75	8	82	84	23
Rutherford 5	235	385	118	70	252	181	161	483	94	426	274	38	331	315	92
Rutherford 6	54	85	39	17	60	47	44	109	25	113	61	4	64	84	30
Total	384	542	179	96	360	265	254	717	134	645	410	50	477	483	145

Blood pressure management good: diabetes or renal failure (−) <140/90 mmHg (+) <130/80 mmHg. Diabetes management good: HbA1c<7.0% (NGSP). Dyslipidemia management good: other sclerotic lesions (−) LDL<100 mg/DL, (+) LDL<80 mg/DL. HbA1c: hemoglobin A1c, LDL: low-density lipoprotein, NGSP: national glycohemoglobin standardization program

**Table:** Table 2-3 Patients’ background 3

a. Total														
	Ischemic heart disease				Heart failure		Cerebrovascular disease		Renal dysfunction					
	(−)	(+)			(−)	(+)	(−)	(+)	(−)	(+)				
		Medical treatment	PCI	CABG						G3a	G3b	G4	G5	G5D
Rutherford 4	129	12	35	23	190	9	161	38	82	16	14	6	1	80
Rutherford 5	484	64	133	80	644	117	600	161	225	81	68	31	11	345
Rutherford 6	114	16	27	28	148	37	138	47	71	15	7	2	2	88
Total	727	92	195	131	982	163	899	246	378	112	89	39	14	513
b. ASO														
	Ischemic heart disease				Heart failure		Cerebrovascular disease		Renal dysfunction					
	(−)	(+)			(−)	(+)	(−)	(+)	(−)	(+)				
		Medical treatment	PCI	CABG						G3a	G3b	G4	G5	G5D
Rutherford 4	119	12	35	23	180	9	152	37	73	16	13	6	1	80
Rutherford 5	466	61	131	80	625	113	580	158	214	74	66	31	11	342
Rutherford 6	108	16	27	27	143	35	133	45	67	15	7	2	2	85
Total	693	89	193	130	948	157	865	240	354	105	86	39	14	507

PCI: percutaneous coronary intervention, CABG: coronary arterial bypass grafting Heart failure (+): history of admission due to heart failure, clinical symptoms due to heart failure confirmed by ultrasound examination, apparently decreased cardiac function by ultrasound examination without clinical symptoms. Renal dysfunction: (−) (60≦), G3a (45～59), G3b (30～44), G4 (15～29), G5 (<15), G5D (<15 with hemodialyais). New CKD risk stratification by eGFR(mL/min/1.73 m^2^) in “Clinical Practice Guidebook for Diagnosis and Treatment of Chronic Kidney Disease 2012” eGFR: estimated glomerular filtration rate, CKD: chronic kidney disease

**Table:** Table 2-4 Patients’ background 4

a. Total															
	Malignant neoplasm				Sites of malignant neoplasm										
	(−)	(+)			Head and neck	Esophagus	Lung	Stomach	Hepatobiliary pancreas	Colon	Breast	Uterus	Ovarium	Prostate	Others
		History of cancer	Under treatment*	Unknown											
Rutherford 4	183	11	5	0	1	1	2	3	1	4	0	1	0	0	3
Rutherford 5	690	56	12	3	4	0	11	12	8	17	3	1	1	4	16
Rutherford 6	167	15	3	0	2	0	3	7	0	0	1	1	0	1	4
Total	1040	82	20	3	7	1	16	22	9	21	4	3	1	5	23
b. ASO															
	Malignant neoplasm				Sites of malignant neoplasm										
	(−)	(+)			Head and neck	Esophagus	Lung	Stomach	Hepatobiliary pancreas	Colon	Breast	Uterus	Ovarium	Prostate	Others
		History of cancer	Under treatment*	Unknown											
Rutherford 4	173	11	5	0	1	1	2	3	1	4	0	1	0	0	3
Rutherford 5	671	52	12	3	4	0	11	11	5	17	3	0	1	4	16
Rutherford 6	160	15	3	0	2	0	3	7	0	0	1	1	0	1	4
Total	1004	78	20	3	7	1	16	21	6	21	4	2	1	5	23

*including palliative therapy or recurrence

**Table:** Table 2-5 Patients’ background 5

a. Total																			
	Contralateral limb occlusive lesions													Vascular lesions excluding occlusion					
	(−)	(+)																	
		Asymptomatic	Intermittent claudication	CLI			Post-treatment	ABI		TBI		SPP		(−)	TAA	AAA (including IAA)	Peripheral artery aneurysm	Carotid stenosis	Others
				R4	R5	R6		n	Median	n	Median	n	Median						
Rutherford 4	59	39	21	37	4	0	39	150	0.85	21	0.50	50	39.5	172	1	10	1	6	9
Rutherford 5	167	231	41	14	152	5	151	552	0.795	75	0.41	315	38.0	694	7	14	1	27	18
Rutherford 6	45	51	10	5	17	24	33	119	0.85	15	0.57	69	41.0	172	1	6	0	4	2
Total	271	321	72	56	173	29	223	821	0.81	111	0.43	434	38.0	1038	9	30	2	37	29
b. ASO																			
	Contralateral limb occlusive lesions													Vascular lesions excluding occlusion					
	(−)	(+)																	
		Asymptomatic	Intermittent claudication	CLI			Post-treatment	ABI		TBI		SPP		(−)	TAA	AAA (including IAA)	Peripheral artery aneurysm	Carotid stenosis	Others
				R4	R5	R6		n	Median	n	Median	n	Median						
Rutherford 4	54	38	19	36	4	0	38	142	0.845	21	0.50	49	39.0	165	1	9	1	6	7
Rutherford 5	161	222	41	14	147	5	148	534	0.79	74	0.41	306	38.0	675	7	12	1	26	17
Rutherford 6	43	49	10	4	15	24	33	113	0.83	15	0.57	65	41.0	167	1	5	0	4	1
Total	258	309	70	54	166	29	219	789	0.8	110	0.43	420	38.0	1007	9	26	2	36	25

ABI: ankle brachial (pressure) index, TBI: toe brachial (pressure) index, SPP: skin perfusion pressure, CLI: critical limb ischemia, TAA: thoracic aortic aneurysm, AAA: abdominal aortic aneurysm, IAA: iliac artery aneurysm

**Table:** Table 2-6 Patients’ background 6

a. Total								
	Fatty acid							
	Arachidonic acid (AA)		Eicosapentaenoic acid (EPA)		Docosahexaenoic acid (DHA)		EPA/AA	
	n	Median	n	Median	n	Median	n	Median
Rutherford 4	6	253.1	6	94.0	6	133.1	6	0.4
Rutherford 5	21	159.8	21	59.6	21	110.6	21	0.4
Rutherford 6	9	159.0	9	31.9	9	75.0	9	0.3
Total	36	160.9	36	49.7	36	97.9	36	0.3
b. ASO								
	Fatty acid							
	Arachidonic acid (AA)		Eicosapentaenoic acid (EPA)		Docosahexaenoic acid (DHA)		EPA/AA	
	n	Median	n	Median	n	Median	n	Median
Rutherford 4	5	273.5	5	143.9	5	143.5	5	0.5
Rutherford 5	20	160.0	20	59.6	20	106.6	20	0.4
Rutherford 6	8	156.8	8	36.1	8	66.1	8	0.3
Total	33	162.0	33	51.1	33	98.3	33	0.3

The causes of the arterial occlusion of the limb were ASO in 1,105 (97%) limbs, thromboangiitis obliterans (TAO) in 4, vasculitis (Takayasu’s arteritis, collagen disease, Behçet’s disease, and fibromuscular dysplasia excluding TAO) in 19, and others in 17. Patients’ comorbidities consisted of diabetes in 65% (65%) of the limbs, hypertension in 76% (77%), dyslipidemia in 41% (42%), ischemic heart disease in 37% (37%), cerebrovascular disease in 21% (22%), dialysis for renal failure in 45% (46%), medical history of malignant neoplasm or that being treated in 9% (9%), and arterial occlusive lesions in the contralateral limb in 76% (77%) and smoking (ex- and current) in 57% (57%).

### (2) Conditions of limb ischemia

Limb ischemia pretreatment conditions are shown in **Tables 3-1** to **3-6**. Regarding the walking function (Taylor classification),^9)^ patients who could walk outdoors or indoors independently, including with a cane, were regarded as “ambulatory,” and those unable to walk but able to stand on their own legs during transfer from the bed to a wheel chair were designated as “ambulatory/homebound.”

Regarding the state of local tissue defect (Texas University classification),^10)^ the most severe lesion, the main treatment target, was evaluated. Skin perfusion pressure (SPP) was measured on the foot (base of the toe, dorsum of the foot, or sole), and a lower value was adopted. To perform the WIfI classification, the sites of ulcer and gangrene were registered separately. Although SPP is widely used as an objective index for evaluating ischemia in Japan, ischemic grading criteria using SPP is not shown in the WIfI classification, in which TP is given top priority. Therefore, in the JCLIMB, the SPP value was converted to TP using the conversion equation SPP=0.6853×TP +14.48 from the correlation data of SPP and TP reported in Japan^11)^ and applied for WIfI ischemic grading (**Table 1-2-2**).

**Table:** Table 3 Pretreatment condition Table 3-1 Pretreatment condition 1

a. Total																										
	Ambulatory function (Taylor’s classification)			Sites of ulcer							Depth of ulcer (University of Texas classification: grade)			Sites of gangrene							Main sites of ulcer/gangrene to be treated					
	Ambulatory	Amburatory/homebound	Nonambulatory	Digits	Foot: distal metatarsal	Foot: proximal metatarsal	Heel	Ankle	Lower leg	Only gangrene w/o ulcer	I	II	III	Digits	Foot: distal metatarsal	Foot: proximal metatarsal	Heel	Ankle	Lower leg	Only gangrene w/o ulcer	Toe	Foot: distal metatarsal	Foot: proximal metatarsal	Heel	Ankle	Lower leg
Rutherford 4	151	28	20																							
Rutherford 5	435	167	159	574	86	18	76	16	19	63	438	133	190	375	58	8	33	5	8	324	594	76	14	54	10	13
Rutherford 6	52	58	75	51	58	32	38	13	30	30	45	48	92	62	64	34	28	8	23	26	43	50	33	24	6	29
Total	638	253	254	625	144	50	114	29	49	93	483	181	282	437	122	42	61	13	31	350	637	126	47	78	16	42
b. ASO																										
	Ambulatory function (Taylor’s classification)			Sites of ulcer							Depth of ulcer (University of Texas classification: grade)			Sites of gangrene							Main sites of ulcer/gangrene to be treated					
	Ambulatory	Amburatory/homebound	Nonambulatory	Digits	Foot: distal metatarsal	Foot: proximal metatarsal	Heel	Ankle	Lower leg	Only gangrene w/o ulcer	I	II	III	Digits	Foot: distal metatarsal	Foot: proximal metatarsal	Heel	Ankle	Lower leg	Only gangrene w/o ulcer	Toe	Foot: distal metatarsal	Foot: proximal metatarsal	Heel	Ankle	Lower leg
Rutherford 4	143	27	19																							
Rutherford 5	423	158	157	562	76	15	74	16	19	61	426	130	182	361	54	7	33	5	8	317	581	69	12	53	10	13
Rutherford 6	50	53	75	49	55	30	38	13	30	30	45	45	88	59	61	32	28	8	23	26	41	47	31	24	6	29
Total	616	238	251	611	131	45	112	29	49	91	471	175	270	420	115	39	61	13	31	343	622	116	43	77	16	42

University of Texas classification: grade (I: superficial, not involving tendon, capsule, or bone, II: penetrating to tendon/capsule, III: penetrating to bone or joint)

**Table:** Table 3-2 Pretreatment condition 2

a. Total																								
	Temperature ≧38°		Blood test								Hemodynamics								Infection*^1)^					
	(−)	(+)	WBC		CRP		Alb		Cr		ABI		TBI		SPP		Toe pressure		Local (foot)				Systemic	
			n	Median	n	Median	n	Median	n	Median	n	Median	n	Median	n	Median	n	Median	Uninfected	Skin or subcutaneous tissue (erythema)*^2)^		Deep tissue*^3)^	SIRS*^4)^	
																				≦2.0 cm	>2.0 cm		(+)	(−)
Rutherford 4	193	6	196	6,550	193	0.37	183	3.60	194	1.45	118	1	12	0.43	69	16.00	12	64.00	178	14	5	2	2	197
Rutherford 5	735	26	737	6,800	722	0.99	701	3.30	737	1.68	476	1	43	0.18	456	24.00	43	23.00	496	171	32	62	13	748
Rutherford 6	165	20	180	8,095	178	4.62	175	2.90	180	1.70	88	1	7	0.27	102	21.50	7	25.00	63	41	25	56	15	170
Total	1093	52	1113	6,950	1093	1.01	1059	3.30	1111	1.63	682	1	62	0.21	627	23.00	62	25.50	737	226	62	120	30	1115
b. ASO																								
	Temperature ≧38°		Blood test								Hemodynamics								Infection*^1)^					
	(−)	(+)	WBC		CRP		Alb		Cr		ABI		TBI		SPP		Toe pressure		Local (foot)				Systemic	
			n	Median	n	Median	n	Median	n	Median	n	Median	n	Median	n	Median	n	Median	Uninfected	Skin or subcutaneous tissue (erythema)*^2)^		Deep tissue*^3)^	SIRS*^4)^	
																				≦2.0 cm	>2.0 cm		(+)	(−)
Rutherford 4	183	6	186	6,520	183	0.36	175	3.60	184	1.50	114	1	12	0.43	67	16.00	12	64.00	169	14	5	1	2	187
Rutherford 5	713	25	714	6,800	699	0.98	679	3.40	714	1.88	460	1	43	0.18	442	24.00	43	23.00	482	163	31	62	12	726
Rutherford 6	160	18	174	8,095	171	4.36	168	2.90	173	1.73	84	1	7	0.27	97	21.00	7	25.00	61	39	24	54	13	165
Total	1056	49	1074	6,920	1053	1.00	1022	3.30	1071	1.74	658	1	62	0.21	606	23.00	62	25.50	712	216	60	117	27	1078

WBC: white blood cell, CRP: C reactive protein, Alb: albumin, Cr: creatinine, ABI: ankle brachial (pressure) index, TBI: toe brachial (pressure) index, SPP: skin perfusion pressure, SIRS: systemic inflammatory response syndrome. *^1)^ Presence of infection is defined by the presence of at least 2 of the following items: ①Local swelling or induration, ②Erythema >0.5 to ≦2 cm around the ulcer, ③Local tenderness or pain, ④Local warmth, ⑤Purulent discharge (thick, opaque to white, or sanguineous secretion). *^2)^ Local infection at skin and subcutaneous tissue was classified by the spreading of erythema (≦2.0 cm or >2 cm) around the ulcer/gangrene. *^3)^ Local infection involving structures deeper than skin and subcutaneous tissues (e.g., abscess, osteomyelitis, septic arthritis, fasciitis). *^4)^ The signs of SIRS are manifested by two or more of the following: ①Temperature >38 or <36°C, ②Heart rate >90 beats/min, ③Respiratory rate >20 breaths/min or PaCO_2_ <32 mmHg, ④White blood cell count >12,000 or <4000 cu/mm or 10% immature (band) forms.

**Table:** Table 3-3 Pretreatment condition 3

a. Total																
	Diagnostic imaging			Sites of occlusion			TASC II classification aortoiliac					TASC II classification femoropopliteal				
	IADSA	CTA	Others	Aortoiliac	Femoropop	Lower leg/foot	A	B	C	D	No lesion	A	B	C	D	No lesion
Rutherford 4	104	114	17	62	125	79	14	12	12	21	0	9	19	26	82	16
Rutherford 5	517	366	17	132	451	494	41	25	18	41	6	76	100	124	243	153
Rutherford 6	118	91	9	34	118	130	10	2	3	12	2	23	17	28	61	38
Total	739	571	43	228	694	703	65	39	33	74	8	108	136	178	386	207
b. ASO																
	Diagnostic imaging			Sites of occlusion			TASC II classification aortoiliac					TASC II classification femoropopliteal				
	IADSA	CTA	Others	Aortoiliac	Femoropop	Lower leg/foot	A	B	C	D	No lesion	A	B	C	D	No lesion
Rutherford 4	99	108	16	59	121	76	14	12	11	19	0	9	19	25	79	15
Rutherford 5	501	351	17	127	437	479	40	24	18	39	6	75	98	124	230	148
Rutherford 6	114	89	8	34	117	123	10	2	3	12	2	22	17	27	60	34
Total	714	548	41	220	675	678	64	38	32	70	8	106	134	176	369	197

IADSA: intra-arterial digital subtraction angiography, CTA: computed tomography angiography

**Table:** Table 3-4 Pretreatment condition 4

a. Total														
	Bollinger Score													
	Common femoral		Deep femoral		Superficial femoral: proximal		Superficial femoral: distal		Popliteal: proximal		Popliteal: distal		Tibioperoneal trunk	
	n	Median	n	Median	n	Median	n	Median	n	Median	n	Median	n	Median
Rutherford 4	79	2	79	2	80	5.5	80	6	80	3	80	3	77	3
Rutherford 5	457	1	458	1	458	3	459	4	459	2	460	2	452	2.5
Rutherford 6	100	1	100	1	100	3	100	4	100	3	100	3	100	3
Total	636	1	637	1	638	3	639	4	639	3	640	2	629	3
b. ASO														
	Bollinger Score													
	Common femoral		Deep femoral		Superficial femoral: proximal		Superficial femoral: distal		Popliteal: proximal		Popliteal: distal		Tibioperoneal trunk	
	n	Median	n	Median	n	Median	n	Median	n	Median	n	Median	n	Median
Rutherford 4	79	2	79	2	80	5.5	80	6	80	3	80	3	77	3
Rutherford 5	444	1	445	1	445	3	446	4	446	2	447	2	440	2
Rutherford 6	96	1	96	1	96	3	96	4	96	3	96	3	96	3
Total	619	1	620	1	621	3	622	4	622	3	623	2	613	3

**Table:** Table 3-5 Pretreatment condition 5

a. Total														
	Bollinger Score													
	Posterior tibial: proximal		Posterior tibial: distal		Anterior tibial: proximal		Anterior tibial: distal		Peroneal: proximal		Peroneal: distal		Foot	
	n	Median	n	Median	n	Median	n	Median	n	Median	n	Median	n	Median
Rutherford 4	77	13	77	13	77	13	77	13	75	6	75	6	57	6
Rutherford 5	448	13	445	13	452	13	444	13	450	6	444	6	392	13
Rutherford 6	96	13	93	13	98	13	94	13	98	5.5	95	6	80	13
Total	621	13	615	13	627	13	615	13	623	6	614	6	529	13
b. ASO														
	Bollinger Score													
	Posterior tibial: proximal		Posterior tibial: distal		Anterior tibial: proximal		Anterior tibial: distal		Peroneal: proximal		Peroneal: distal		Foot	
	n	Median	n	Median	n	Median	n	Median	n	Median	n	Median	n	Median
Rutherford 4	77	13	77	13	77	13	77	13	75	6	75	6	57	6
Rutherford 5	436	13	433	13	441	13	433	13	438	6	432	6	381	13
Rutherford 6	93	13	90	13	94	13	91	13	95	5	92	6	77	13
Total	606	13	600	13	612	13	601	13	608	6	599	6	515	13

**Table:** Table 3-6 SVS WIfI classification

a. Total																
	Wound				Ischemia				Foot infection				Stage			
	0	1	2	3	0	1	2	3	0	1	2	3	1	2	3	4
Rutherford 4	199	0	0	0	23	29	18	77	178	12	7	2	51	85	10	1
Rutherford 5	0	270	368	123	62	105	69	377	496	169	85	11	41	66	208	298
Rutherford 6	0	12	39	134	12	18	14	89	63	39	70	13	4	3	12	114
Total	199	282	407	257	97	152	101	543	737	220	162	26	96	154	230	413
b. ASO																
	Wound				Ischemia				Foot infection				Stage			
	0	1	2	3	0	1	2	3	0	1	2	3	1	2	3	4
Rutherford 4	189	0	0	0	22	27	18	75	169	12	6	2	48	83	10	1
Rutherford 5	0	266	355	117	60	101	69	365	482	161	84	11	39	65	204	287
Rutherford 6	0	12	37	129	11	18	11	87	61	37	68	12	4	3	12	108
Total	189	278	392	246	93	146	98	527	712	210	158	25	91	151	226	396

The lesion was considered infected when it showed two or more of the following findings: local swelling or induration, erythema >0.5 cm around the ulcer, local tenderness or pain, local warmth, and purulent discharge (thick, opaque to white, or sanguineous secretion). In addition, local infections involving only the skin and the subcutaneous tissue, and those involving structures deeper than the skin and subcutaneous tissues, were registered separately. Local infections involving only the skin and the subcutaneous tissue were differentiated based on the size of the erythema around the ulcer, ≦2 or >2 cm.

Systemic inflammatory response syndrome, indicating systemic infection, was manifested by two or more of the following signs: temperature >38°C or <36°C, heart rate >90 beats/min, respiratory rate >20 breaths/min or PaCO_2_ <32 mmHg, or white blood cell count >12,000 or <4,000 cu/mm or 10% immature (band) forms. The arteries in the ankle joint region were classified as foot arteries.

In pretreatment, 56% (56%) of the patients were ambulatory, 22% (22%) were ambulatory/homebound, and 22% (22%) were non-ambulatory. On the Rutherford classification (R),^12)^ the limbs with categories R4, R5, and R6 accounted for 17% (17%), 66% (67%), and 16% (16%) of the limbs, respectively. The median ankle brachial index (ABI), the toe brachial index, and the SPP of the measured limbs were 0.65 (0.65), 0.21 (0.21), and 23 mmHg (23 mmHg), respectively. The occlusive lesion was located in the aortoiliac artery in 20% (20%) of the limbs, in the femoropopliteal artery in 61% (61%) of the limbs, and in the crural or foot artery in 61% (61%) of the limbs. The multiple occlusive lesions were located in the aortoiliac artery and the femoropopliteal artery in 11% (11%) of limbs, in the aortoiliac artery and the crural or foot artery in 6% (6%), in the femoropopliteal artery and the crural or foot artery in 30% (30%), and in the aortoiliac artery, the femoropopliteal artery, and the crural or foot artery in 5% (5%).

We were able to apply the WIfI classification with sufficient data to 892 limbs (864 limbs). On the WIfI classification, the limbs with stages 1, 2, 3, and 4 accounted for 11% (11%), 17% (17%), 26% (26%), and 46% (46%) of the limbs, respectively.

The problems and considerations on these spreadsheets are described below. In **Table 3-3**, the total number of limbs in the TASC II classification differed compared with the number in each column of the site of occlusion. In the “aortoiliac” lesion, a decreased number of that in the TASC II classification may have been due to input omission. In the “femoropopliteal” lesion, an increased number of that in the TASC II classification may have been due to including the crural lesions.

In **Table 3-6**, there was some dissociation between the R and Wound grades. This may be because of the R grade’s obscure definition. For example, extensive gangrene involving the forefoot is classified in R5 and W3, whereas a shallow ulcer without exposure of the distal leg bone is classified in R6 and W1.

In **Table 3-6**, 97 limbs (93 limbs) were registered as Ischemic grade 0 in the WIfI classification. By definition, a limb with Ischemic grade 0 has a TP of 60 mmHg or more (SPP 56 mmHg or more in the JCLIMB) or AP higher than 100 mmHg, or if arterial calcification precludes reliable AP or TP measurements, TcPO_2_ 60 mmHg or more (**Table 1-1-2**). There should be no limb with Ischemic grade 0 since CLI registered in the JCLIMB is defined according to the TASC II classification. The limbs might be clinically judged to be CLI irrespective of the objective ischemic index, although details are unknown.

In **Table 3-6**, there were 21 limbs (20 limbs) in which infection was confirmed in R4 limbs, despite the absence of a local wound by definition of R4. This may occur because tissue loss is not always requisite for fI grade.

In **Table 3-6**, because ischemic grade data were registered in only 893 limbs (864 limbs) among 1,145 limbs (1,105 limbs), the WIfI classification could be implemented for these 893 limbs (864 limbs). The limbs clinically judged to be CLI could be registered without their objective ischemic index.

### (3) Treatment

**Tables 4-1** to **4-6** show the CLI treatment data. Revascularizations of the affected limbs were performed in 96% (96%) of the registered limbs, and primary major amputations were performed in 1.7% (1.5%) of the registered limbs. Among the surgical reconstruction procedures, distal bypass accounted for 50% (49%). Endovascular treatment (EVT), including EVT alone and hybrid treatment with surgical reconstruction, accounted for 55% (55%) of the total revascularization procedures. EVT applied to the crural or foot artery accounted for 40% (40%) of the total EVT.

The problems and considerations on these spreadsheets are described below. In **Table 4-1**, the sum of the number of cells in treatment is larger than the sum of the number of registered limbs, 1,145 (1,105), because more than one treatment method can be selected. In **Table 4-1**, the discrepancy in the number of major amputation to the number of detail of amputation was caused by “unused.” In the column of “vein usage” in **Table 4-3**, how the autologous veins were used was described when they were selected as vascular conduits. The sum of the number in the column of vein usage, “in-situ,” “non-reversed,” “reversed,” and “spliced,” is larger than the sum of the number in the column of vein in vascular prosthesis. It could be because of selecting multiple vein usage for arterial reconstruction in a limb. Two veins were used in 10 limbs. Vascular prosthesis (−) included an endarterectomy without a patch angioplasty. In **Table 4-4**, the sum of the number of proximal anastomosis is not equal to the sum of the number of distal anastomosis. This was because multiple arteries could be selected in each anastomosis. The sum of the number of distal (crural/foot) bypass in **Table 4-2** is not equal to the sum of the number of distal anastomosis in **Table 4-4**. This was because multiple anastomosis sites could be selected in distal bypass in **Table 4-4**, though either femoral–crural/foot or popliteal–crural/foot was selected in bypass in **Table 4-2**.

**Table:** Table 4 Treatment Table 4-1 Treatment 1

a. Total																				
	Treatment					Angiogenic therapy			Amputation							Reoperation				
	Pharmacological therapy	Angiogenic therapy	Arterial reconstruction	Major amputation	Lumber sympathectomy	Bone marrow	Peripheral blood	Others	Toe	Metatarsal	Chopart Lisfranc	Syme	Below knee	Above knee·knee disarticulation	Hip disarticulation	Unknown	(−)	(+)		
																		1X	2X	3X≦
Rutherford 4	48	1	184	1	1	0	1	0	1	0	0	0	0	0	0	4	141	35	8	11
Rutherford 5	223	0	740	3	0	0	0	0	12	11	0	0	0	0	0	6	586	120	33	16
Rutherford 6	54	0	172	15	0	0	0	0	2	4	1	0	1	4	0	1	145	28	6	5
Total	325	1	1096	19	1	0	1	0	15	15	1	0	1	4	0	11	872	183	47	32
b. ASO																				
	Treatment					Angiogenic therapy			Amputation							Reoperation				
	Pharmacological therapy	Angiogenic therapy	Arterial reconstruction	Major amputation	Lumber sympathectomy	Bone marrow	Peripheral blood	Others	Toe	Metatarsal	Chopart Lisfranc	Syme	Below knee	Above knee·knee disarticulation	Hip disarticulation	Unknown	(−)	(+)		
																		1X	2X	3X≦
Rutherford 4	46	1	176	0	1	0	1	0	0	0	0	0	0	0	0	4	135	31	8	11
Rutherford 5	214	0	718	3	0	0	0	0	12	10	0	0	0	0	0	6	566	117	33	16
Rutherford 6	52	0	165	14	0	0	0	0	2	4	1	0	1	4	0	1	138	28	6	5
Total	312	1	1059	17	1	0	1	0	14	14	1	0	1	4	0	11	839	176	47	32

**Table:** Table 4-2 Treatment 2

a. Total															
	Bypass											TEA			EVT
	Aorta-aorta	Aorta (with suprarenal clamp)	Aorta-femoral	Femoral-proximal popliteal	Femoral-distal popliteal	Femoral-crural/foot	Popliteal-crural/foot	Anatomical others	Axillary-femoral	Femoral-femoral	Extra-anatomical others	Aorta/iliac	Fomoral/popliteal	Others	
Rutherford 4	1	0	6	15	15	26	14	2	3	10	1	2	20	1	104
Rutherford 5	2	0	3	45	38	80	114	4	4	7	2	2	59	4	475
Rutherford 6	0	0	1	6	11	21	30	0	0	3	2	1	11	0	103
Total	3	0	10	66	64	127	158	6	7	20	5	5	90	5	682
b. ASO															
	Bypass											TEA			EVT
	Aorta-aorta	Aorta (with suprarenal clamp)	Aorta-femoral	Femoral-proximal popliteal	Femoral-distal popliteal	Femoral-crural/foot	Popliteal-crural/foot	Anatomical others	Axillary-femoral	Femoral-femoral	Extra-anatomical others	Aorta/iliac	Fomoral/popliteal	Others	
Rutherford 4	1	0	4	14	15	23	12	2	3	10	1	2	20	1	102
Rutherford 5	1	0	3	45	36	78	108	3	4	7	2	2	59	4	464
Rutherford 6	0	0	1	6	11	21	26	0	0	3	2	1	11	0	100
Total	2	0	8	65	62	122	146	5	7	20	5	5	90	5	666

TEA: thromboendarterectomy, EVT: endovascular treatment/therapy

**Table:** Table 4-3 Treatment 3

a. Total															
	EVT				Vascular prosthesis					Vein usage				Vein quality	
	Aorta/iliac	Fomoral/popliteal	Tibioperoneal/foot	Others	Polyester	ePTFE	Vein	Others	(−)	In-situ	Non-reversed	Reversed	Spliced	Good	Poor
Rutherford 4	42	47	27	3	10	28	66	0	16	13	25	24	5	65	1
Rutherford 5	105	235	258	5	12	53	249	4	22	52	102	79	22	236	13
Rutherford 6	22	50	61	1	1	8	63	1	19	12	26	22	6	55	8
Total	169	332	346	9	23	89	378	5	57	77	153	125	33	356	22
b. ASO															
	EVT				Vascular prosthesis					Vein usage				Vein quality	
	Aorta/iliac	Fomoral/popliteal	Tibioperoneal/foot	Others	Polyester	ePTFE	Vein	Others	(−)	In-situ	Non-reversed	Reversed	Spliced	Good	Poor
Rutherford 4	41	47	26	3	6	28	62	0	14	11	24	23	5	61	1
Rutherford 5	103	230	254	4	10	53	238	4	22	50	97	76	21	225	13
Rutherford 6	22	49	58	1	1	8	60	1	18	12	24	21	6	53	7
Total	166	326	338	8	17	89	360	5	54	73	145	120	32	339	21

**Table:** Table 4-4 Treatment 4

a. Total																			
	Distal bypass																		
	Proximal anastomosis								Distal anastomosis		Distal anastomosis: sites of crural artery				Distal anastomosis: sites of foot artery				
	External iliac	Common femoral	Deep femoral	Superficial femoral	Proximal popliteal	Distal popliteal	Crural	Others	Crural	Foot	Tibioperoneal trunk	Posterior tibial	Anterior tibial	Peroneal	Posterior tibial	Anterior tibial	Peroneal	Dorsalis pedis	Plantar
Rutherford 4	0	14	2	8	6	4	2	1	17	19	2	12	1	2	7	2	1	9	0
Rutherford 5	5	46	6	36	25	89	5	7	79	137	6	34	31	10	22	20	5	78	15
Rutherford 6	0	6	3	6	4	18	3	1	13	28	0	7	4	2	8	2	0	16	3
Total	5	66	11	50	35	111	10	9	109	184	8	53	36	14	37	24	6	103	18
b. ASO																			
	Distal bypass																		
	Proximal anastomosis								Distal anastomosis		Distal anastomosis: sites of crural artery				Distal anastomosis: sites of foot artery				
	External iliac	Common femoral	Deep femoral	Superficial femoral	Proximal popliteal	Distal popliteal	Crural	Others	Crural	Foot	Tibioperoneal trunk	Posterior tibial	Anterior tibial	Peroneal	Posterior tibial	Anterior tibial	Peroneal	Dorsalis pedis	Plantar
Rutherford 4	0	14	2	8	6	3	2	1	17	18	2	12	1	2	6	2	1	9	0
Rutherford 5	5	44	6	35	25	82	5	7	75	131	5	32	31	9	20	20	5	77	12
Rutherford 6	0	6	3	6	4	18	2	1	13	27	0	7	4	2	7	2	0	16	3
Total	5	64	11	49	35	103	9	9	105	176	7	51	36	13	33	24	6	102	15

**Table:** Table 4-5 Treatment 5

a. Total						
	Pharmacological therapy					
	Antiplatelet	VKA	Prostaglandin	Heparin	Statin	Others
Rutherford 4	66	10	5	5	15	3
Rutherford 5	340	35	35	24	44	14
Rutherford 6	75	10	17	11	15	4
Total	481	55	57	40	74	21
b. ASO						
	Pharmacological therapy					
	Antiplatelet	VKA	Prostaglandin	Heparin	Statin	Others
Rutherford 4	65	8	4	4	15	3
Rutherford 5	323	33	31	21	42	11
Rutherford 6	73	9	17	11	13	4
Total	461	50	52	36	70	18

Antiplatelet: aspirin, cilostazol, beraprost, sarpogrelate, ticlopidine, clopidogrel, ethyl icosapentate.

**Table:** Table 4-6 Treatment 6

a. Total				
	Femoral-proximal popliteal bypass	Femoral-distal popliteal bypass	Femoral-crural/foot bypass	Popliteal-crural/foot bypass
Polyester	4	1	1	0
ePTFE	43	12	0	6
Vein	25	50	121	155
Artery	0	2	4	1
Others	0	0	1	0
(−)	0	0	0	2
Total	72	65	127	164
b. ASO				
	Femoral-proximal popliteal bypass	Femoral-distal popliteal bypass	Femoral-crural/foot bypass	Popliteal-crural/foot bypass
Polyester	3	1	0	0
ePTFE	43	12	0	6
Vein	25	48	117	144
Artery	0	2	4	1
Others	0	0	1	0
(−)	0	0	0	1
Total	71	63	122	152

**Table 4-6** summarizes the vascular grafts used for infrainguinal arterial reconstruction. For example, the total number of vascular graft in the column of femoral–proximal popliteal artery bypass was 72 (71), which was higher than 66 (65), the number of actual applications in **Table 4-2**. This was because multiple graft materials could be selected when multiple procedures, such as a bypass procedure and thromboendarterectomy (TEA), can be performed simultaneously for arterial reconstruction in a lower limb. When TEA without patch angioplasty was performed, “unused” was selected.

### (4) Outcomes of early (1 month) after treatment

**Tables 5-1** to **5-8** show the outcomes of early (1 month) after treatment. At the time of summary count at the end of March 2019, follow-up data 1 month after treatment were obtained in 993 limbs (87%), including 956 limbs (87%) with ASO. Data were collected according to the severity of the local limb conditions (Rutherford classification) and treatment measures (EVT alone or surgical reconstruction with/without EVT). The mortality rate was 2.3% (2.3%) in the whole series, and the mortality rates were 2.8% (2.8%) and 1.7% (1.5%) treated by EVT alone and by surgical reconstruction with/without EVT, respectively. The most common cause of death was cardiac disease, which accounted for 22% (23%) of all deaths. Postoperative complications were cardiac disease in 3.4% (3.5%), cerebrovascular disease in 1.3% (1.3%), pneumonia in 1.7% (1.7%), and wound complication in 4.3% (4.3%). Complications at the puncture site were noted in 1.9% (2.0%) of the limbs treated by EVT alone.

**Table:** Table 5 Outcomes early (one month) after treatment therapeutic measures: EVT (only EVT without surgical reconstruction), Surgical reconstruciton (surgical reconstruction with or without EVT) Table 5-1 Life prognosis/causes of death

a. Total																
		Life prognosis			Causes of death											
		Alive	Dead	Unknown	Cardiac disease	Cerebrovascular disease			Malignant neoplasm	Aortic aneurysm/dissection	Infection		Ischemic enteritis	Gastrointestinal bleeding	Others	Unknown
						Hemorrhage	Infarction	Unknown			Diseased limb	Others				
Local condition	Rutherford 4	167	4	0	1	0	0	0	0	0	0	1	0	0	2	0
	Rutherford 5	657	14	0	3	0	0	0	1	0	2	1	2	0	3	2
	Rutherford 6	146	5	0	1	1	0	0	0	0	1	1	0	0	0	1
Therapeutic measures	Non-reconstruction	40	2	0	0	0	0	0	0	0	0	2	0	0	0	0
	EVT	459	13	0	4	1	0	0	1	0	3	1	0	0	1	2
	Surgical reconstruction	471	8	0	1	0	0	0	0	0	0	0	2	0	4	1
Total		970	23	0	5	1	0	0	1	0	3	3	2	0	5	3
b. ASO																
		Life prognosis			Causes of death											
		Alive	Dead	Unknown	Cardiac disease	Cerebrovascular disease			Malignant neoplasm	Aortic aneurysm/dissection	Infection		Ischemic enteritis	Gastrointestinal bleeding	Others	Unknown
						Hemorrhage	Infarction	Unknown			Diseased limb	Others				
Local condition	Rutherford 4	159	4	0	1	0	0	0	0	0	0	1	0	0	2	0
	Rutherford 5	635	13	0	3	0	0	0	1	0	2	1	2	0	2	2
	Rutherford 6	140	5	0	1	1	0	0	0	0	1	1	0	0	0	1
Therapeutic measures	Non-reconstruction	37	2	0	0	0	0	0	0	0	0	2	0	0	0	0
	EVT	448	13	0	4	1	0	0	1	0	3	1	0	0	1	2
	Surgical reconstruction	449	7	0	1	0	0	0	0	0	0	0	2	0	3	1
Total		934	22	0	5	1	0	0	1	0	3	3	2	0	4	3

**Table:** Table 5-2 Perioperative complications 1

a. Total																
		Cardiac disease				Cerebrovascular disease				Pneumonia		Wound complication		Peripheral embolism		
		(−)	Angina	Serious arrhysmia	Myocardial infarction	(−)	TIA	Cerebral infarction		(−)	(+)	(−)	(+)	(−)	(+)	
								Functional loss (−)	Functional loss (+)						Minor (including blue toe)	Major
Local condition	Rutherford 4	152	6	0	0	154	1	0	3	157	1	151	7	157	1	0
	Rutherford 5	628	12	8	5	648	0	3	2	643	10	627	26	647	3	3
	Rutherford 6	147	1	1	0	146	0	2	1	144	5	141	8	148	1	0
Therapeutic measures	Non-reconstruction	9	0	0	0	9	0	0	0	8	1	9	0	9	0	0
	EVT	457	11	1	3	468	1	0	3	462	10	468	4	467	3	2
	Surgical reconstruction	461	8	8	2	471	0	5	3	474	5	442	37	476	2	1
Total		927	19	9	5	948	1	5	6	944	16	919	41	952	5	3
b. ASO																
		Cardiac disease				Cerebrovascular disease				Pneumonia		Wound complication		Peripheral embolism		
		(−)	Angina	Serious arrhysmia	Myocardial infarction	(−)	TIA	Cerebral infarction		(−)	(+)	(−)	(+)	(−)	(+)	
								Functional loss (−)	Functional loss (+)						Minor (including blue toe)	Major
Local condition	Rutherford 4	146	6	0	0	148	1	0	3	151	1	145	7	151	1	0
	Rutherford 5	607	11	8	5	626	0	3	2	621	10	605	26	625	3	3
	Rutherford 6	141	1	1	0	140	0	2	1	138	5	136	7	142	1	0
Therapeutic measures	Non-reconstruction	9	0	0	0	9	0	0	0	8	1	9	0	9	0	0
	EVT	447	10	1	3	457	1	0	3	451	10	457	4	456	3	2
	Surgical reconstruction	438	8	8	2	448	0	5	3	451	5	420	36	453	2	1
Total		894	18	9	5	914	1	5	6	910	16	886	40	918	5	3

TIA: transient ischemic attack

**Table:** Table 5-3 Perioperative complications 2

a. Total															
		Hemorrhage			Sites of bleeding			Outcome of bleeding				Complication due to contrast medium		Complication at puncture site	
		(−)	(+)	Unknown	Brain	GI tract	Others	Cured	Uncured	Dead	Others	(−)	(+)	(−)	(+)
Local condition	Rutherford 4	155	3	0	0	0	3	3	0	0	0	157	1	88	0
	Rutherford 5	645	8	0	0	4	4	7	1	0	0	653	0	398	8
	Rutherford 6	145	4	0	1	3	0	3	0	1	0	149	0	77	2
Therapeutic measures	Non-reconstruction	8	1	0	0	1	0	1	0	0	0	9	0	15	1
	EVT	467	5	0	1	3	1	4	0	1	0	472	0	463	9
	Surgical reconstruction	470	9	0	0	3	6	8	1	0	0	478	1	85	0
Total		945	15	0	1	7	7	13	1	1	0	959	1	563	10
b. ASO															
		Hemorrhage			Sites of bleeding			Outcome of bleeding				Complication due to contrast medium		Complication at puncture site	
		(−)	(+)	Unknown	Brain	GI tract	Others	Cured	Uncured	Dead	Others	(−)	(+)	(−)	(+)
Local condition	Rutherford 4	149	3	0	0	0	0	3	0	0	0	151	1	86	0
	Rutherford 5	624	7	0	0	4	3	6	1	0	0	631	0	387	8
	Rutherford 6	139	4	0	1	3	3	3	0	1	0	143	0	75	2
Therapeutic measures	Non-reconstruction	8	1	0	0	1	0	1	0	0	0	9	0	14	1
	EVT	456	5	0	1	3	1	4	0	1	0	461	0	452	9
	Surgical reconstruction	448	8	0	0	3	5	7	1	0	0	455	1	82	0
Total		912	14	0	1	7	6	12	1	1	0	925	1	548	10

GI: gastrointestinal

**Table:** Table 5-4 Hemodynamics

a. Total													
		Immediate after the treatment						One month after the treatment					
		ABI		Ankle pressure		SPP		ABI		Ankle pressure		SPP	
		n	Median	n	Median	n	Median	n	Median	n	Median	n	Median
Local condition	Rutherford 4	85	0.84	68	111	39	40	66	0.89	47	107.00	16	45
	Rutherford 5	299	0.93	280	119	271	41	228	0.99	211	131	118	48
	Rutherford 6	47	0.92	45	123	51	41	36	0.90	34	119.5	28	43.5
Therapeutic measures	Non-reconstruction	17	0.88	13	107	10	34.5	12	0.85	7	106	4	45.5
	EVT	230	0.89	211	116	174	40	180	0.97	162	125	87	46
	Surgical reconstruction	184	0.945	169	121	177	42	138	0.97	123	129	71	48
Total		431	0.92	393	118	361	41	330	0.96	292	127	162	47
b. ASO													
		Immediate after the treatment						One month after the treatment					
		ABI		Ankle pressure		SPP		ABI		Ankle pressure		SPP	
		n	Median	n	Median	n	Median	n	Median	n	Median	n	Median
Local condition	Rutherford 4	83	0.84	67	111	38	38.5	64	0.89	46	108.00	15	42
	Rutherford 5	292	0.93	273	119	264	41	223	0.99	208	130	115	48
	Rutherford 6	44	0.92	42	114.5	49	41	35	0.89	33	119	26	43.5
Therapeutic measures	Non-reconstruction	16	0.93	12	109	9	31	11	0.82	6	115.5	3	41
	EVT	225	0.89	206	115	170	40	177	0.95	160	125	85	46
	Surgical reconstruction	178	0.95	164	121	172	42	134	0.97	121	129	68	48
Total		419	0.92	382	118	351	41	322	0.96	287	126	156	47

ABI: ankle brachial (pressure) index, SPP: skin perfusion pressure

**Table:** Table 5-5 Condition of the limbs

a. Total																		
		Bypass graft/EVT condition							Clinical symptoms of the limb			Ischemic wound				Ambulatory function at discharge (Taylor's classification)		
		Good	Stenosis	Occlusion	Deterioration	Anastomosis disruption (aneurysm)	Infection	Others	Improved	No change	Deteriorated	Cured	Uncured		Unknown	Ambulatory	Amburatory/homebound	Nonambulatory
													Improved	Unchanged/deteriorated				
Local condition	Rutherford 4	146	3	7	0	1	0	1	143	21	5	115	35	18	1	119	21	31
	Rutherford 5	602	9	31	0	1	4	9	517	117	29	141	377	137	8	339	146	186
	Rutherford 6	126	3	9	0	1	1	4	113	21	8	19	94	29	0	40	48	63
Therapeutic measures	Non-reconstruction	0	0	0	0	0	0	0	19	6	1	11	9	5	1	22	4	16
	EVT	429	13	19	0	0	3	11	352	92	28	118	232	118	4	217	93	162
	Surgical reconstruction	445	2	28	0	3	2	3	402	61	13	146	265	61	4	259	118	102
Total		874	15	47	0	3	5	14	773	159	42	275	506	184	9	498	215	280
b. ASO																		
		Bypass graft/EVT condition							Clinical symptoms of the limb			Ischemic wound				Ambulatory function at discharge (Taylor's classification)		
		Good	Stenosis	Occlusion	Deterioration	Anastomosis disruption (aneurysm)	Infection	Others	Improved	No change	Deteriorated	Cured	Uncured		Unknown	Ambulatory	Amburatory/homebound	Nonambulatory
													Improved	Unchanged/deteriorated				
Local condition	Rutherford 4	140	3	7	0	1	0	1	138	19	5	110	34	17	1	113	20	30
	Rutherford 5	585	9	27	0	1	4	7	500	114	27	138	362	133	8	326	137	185
	Rutherford 6	122	2	8	0	1	1	4	108	20	8	19	89	28	0	38	44	63
Therapeutic measures	Non-reconstruction	0	0	0	0	0	0	0	18	5	1	9	9	5	1	20	3	16
	EVT	422	12	17	0	0	3	10	345	90	26	116	227	114	4	211	88	162
	Surgical reconstruction	425	2	25	0	3	2	2	383	58	13	142	249	59	4	246	110	100
Total		847	14	42	0	3	5	12	746	153	40	267	485	178	9	477	201	278

**Table:** Table 5-6 Revision of treatment

a. Total																		
		Revision for those excluding good bypass graft/EVT condition		Minor reintervention (revision for stenosis)				Major reintervention (revision for occlusion)								Major amputation		
		(+)	(−)	(−)	Patch plasty	EVT	Others	(−)	Thrombectomy (±patch plasty)	Thrombolysis	EVT	Re-bypass	Jump bypass	Interposition	Others	(−)	(+)	
																	Due to preoperative wound	Due to new wound
Local condition	Rutherford 4	6	6	155	0	2	0	154	0	0	1	2	0	0	0	163	4	3
	Rutherford 5	23	30	635	1	13	2	618	5	0	6	9	11	0	2	650	17	2
	Rutherford 6	8	8	136	0	4	0	128	3	0	1	4	1	1	2	129	13	0
Therapeutic measures	Non-reconstruction	0	0	0	0	0	0	0	0	0	0	0	0	0	0	30	3	0
	EVT	16	28	458	0	13	1	447	2	0	5	8	7	1	2	444	25	3
	Surgical reconstruction	21	16	468	1	6	1	453	6	0	3	7	5	0	2	468	6	2
Total		37	44	926	1	19	2	900	8	0	8	15	12	1	4	942	34	5
b. ASO																		
		Revision for those excluding good bypass graft/EVT condition		Minor reintervention (revision for stenosis)				Major reintervention (revision for occlusion)								Major amputation		
		(+)	(−)	(−)	Patch plasty	EVT	Others	(−)	Thrombectomy (±patch plasty)	Thrombolysis	EVT	Re-bypass	Jump bypass	Interposition	Others	(−)	(+)	
																	Due to preoperative wound	Due to new wound
Local condition	Rutherford 4	6	6	150	0	2	0	149	0	0	1	2	0	0	0	157	3	3
	Rutherford 5	20	27	614	1	12	2	599	5	0	5	8	10	0	2	628	16	2
	Rutherford 6	7	7	130	0	4	0	123	2	0	1	4	1	1	2	124	12	0
Therapeutic measures	Non-reconstruction	0	0	0	0	0	0	0	0	0	0	0	0	0	0	28	2	0
	EVT	16	24	448	0	12	1	437	2	0	5	8	6	1	2	435	23	3
	Surgical reconstruction	17	16	446	1	6	1	434	5	0	2	6	5	0	2	446	6	2
Total		33	40	894	1	18	2	871	7	0	7	14	11	1	4	909	31	5

**Table:** Table 5-7 Condition of contralateral limbs

a. Total																		
		Contralateral limb occlusive lesions							Treatment for contralateral limb									
		(−)	(+)						Unnecessary	(+)								
			Asymptomatic	Intermittent claudication	CLI			Post-treatment		Pharmacological therapy	Angiogenic therapy	EVT	Surgical bypass	Minor amputation	Major amputation	Lumber sympathectomy	Necessary but no treatment	Others
					R4	R5	R6											
Local condition	Rutherford 4	68	40	15	7	3	1	37	4	66	0	17	21	0	2	0	3	1
	Rutherford 5	208	254	32	8	53	2	114	57	315	0	79	44	13	24	0	7	2
	Rutherford 6	47	47	5	2	13	5	32	14	64	0	15	18	4	6	0	1	2
Therapeutic measures	Non-reconstruction	15	10	0	2	1	0	14	5	12	0	6	2	1	3	0	1	0
	EVT	159	155	17	2	44	4	91	35	213	0	77	25	10	18	0	5	3
	Surgical reconstruction	149	176	35	13	24	4	78	35	220	0	28	56	6	11	0	5	2
Total		323	341	52	17	69	8	183	75	445	0	111	83	17	32	0	11	5
b. ASO																		
		Contralateral limb occlusive lesions							Treatment for contralateral limb									
		(−)	(+)						Unnecessary	(+)								
			Asymptomatic	Intermittent claudication	CLI			Post-treatment		Pharmacological therapy	Angiogenic therapy	EVT	Surgical bypass	Minor amputation	Major amputation	Lumber sympathectomy	Necessary but no treatment	Others
					R4	R5	R6											
Local condition	Rutherford 4	64	40	14	6	3	1	35	4	66	0	16	18	0	2	0	3	0
	Rutherford 5	199	246	32	8	52	1	110	55	304	0	75	43	12	24	0	7	2
	Rutherford 6	43	45	5	2	13	5	32	14	62	0	15	18	4	6	0	1	2
Therapeutic measures	Non-reconstruction	14	9	0	2	1	0	13	4	12	0	5	1	1	3	0	1	0
	EVT	153	153	17	2	43	3	90	35	208	0	75	25	10	18	0	5	3
	Surgical reconstruction	139	169	34	12	24	4	74	34	212	0	26	53	5	11	0	5	1
Total		306	331	51	16	68	7	177	73	432	0	106	79	16	32	0	11	4

CLI: critical limb ischemia

**Table:** Table 5-8 Malignant neoplasm

a. Total															
		Newly diagnosed malignant neoplasm			Sites of newly diagnosed malignant neoplasm										
		(−)	(+)	Unknown	Head and neck	Esophagus	Lung	Stomach	Hepatobiliary pancreas	Colon	Breast	Uterus	Ovarium	Prostate	Others
Local condition	Rutherford 4	169	0	2	0	0	0	0	0	0	0	0	0	0	0
	Rutherford 5	661	0	10	0	0	0	0	0	0	0	0	0	0	0
	Rutherford 6	148	1	2	1	0	0	0	0	0	0	0	0	0	0
Therapeutic measures	Non-reconstruction	41	0	1	0	0	0	0	0	0	0	0	0	0	0
	EVT	469	0	3	0	0	0	0	0	0	0	0	0	0	0
	Surgical reconstruction	468	1	10	1	0	0	0	0	0	0	0	0	0	0
Total		978	1	14	1	0	0	0	0	0	0	0	0	0	0
b. ASO															
		Newly diagnosed malignant neoplasm			Sites of newly diagnosed malignant neoplasm										
		(−)	(+)	Unknown	Head and neck	Esophagus	Lung	Stomach	Hepatobiliary pancreas	Colon	Breast	Uterus	Ovarium	Prostate	Others
Local condition	Rutherford 4	161	0	2	0	0	0	0	0	0	0	0	0	0	0
	Rutherford 5	639	0	9	0	0	0	0	0	0	0	0	0	0	0
	Rutherford 6	142	1	2	1	0	0	0	0	0	0	0	0	0	0
Therapeutic measures	Non-reconstruction	38	0	1	0	0	0	0	0	0	0	0	0	0	0
	EVT	459	0	2	0	0	0	0	0	0	0	0	0	0	0
	Surgical reconstruction	445	1	10	1	0	0	0	0	0	0	0	0	0	0
Total		942	1	13	1	0	0	0	0	0	0	0	0	0	0

The median ABI and SPP of the measured limbs, immediately after treatment and 1 month after treatment, were 0.92 (0.92) and 0.96 (0.96) and 41 (41) mmHg and 47 (47) mmHg, respectively. Stenosis, occlusion, infection, or other trouble occurred after revascularization by EVT alone in 9.7% (9.1%) and by surgical reconstruction with/without EVT in 7.9% (7.4%). Secondary major amputation rates were 5.9% (5.6%) in EVT alone and 1.7% (1.8%) in surgical reconstruction with/without EVT. When ambulatory function at discharge was compared with that before surgery, the rate of patients with ambulatory changed from 56% (56%) to 50% (50%), ambulatory/homebound from 22% (22%) to 22% (21%), and non-ambulatory from 22% (23%) to 28% (29%).

The problems, comments, and considerations on these spreadsheets are described below. The number of “bypass graft/EVT condition,” “clinical limb symptoms,” “ischemic wound,” and “ambulatory function at discharge” did not match (**Table 5-5**). The total number of “ambulatory function at discharge” was 993 (956), which was equal to the number of life prognoses (**Table 5-1**), indicating no “unused.” The number of “bypass graft/EVT condition” was not equal to the number of “ambulatory function at discharge” because the objectives of “bypass graft/EVT condition” were the limbs of survivors with arterial reconstruction and because more than one condition could be selected. The number of “clinical symptoms of limb” and “ischemic wound” was not identical. They must be identical because their objectives were survivor without major amputations. This is speculated to be due to the presence of “unused.” The discrepancy in the total number of “life prognosis,” “clinical limb symptom,” and “amputation” is due to the difference of condition for aggregation of data. In **Table 5-3**, the registration of complication at the puncture site in non-reconstruction appears to be odd. The registration of complication at the puncture site was required in the limbs where PTA/STENT was selected in the revascularization method. Since multiple treatment methods can be selected, complications at the puncture site was registered in non-reconstruction and surgical reconstruction.

The number of the limbs of survivors with EVT was 459 (448 limbs) (**Table 5-1**), which was 13 (13) limbs less than the sum of the number in the column of minor reintervention or major reintervention in the row of the limbs with EVT; 472 limbs (461 limbs) (**Table 5-6**). The number of the limbs of survivors with surgical reconstruction was 471 (449 limbs) (**Table 5-1**), which was 5 (5) limbs less than the sum of the number in the column of minor reintervention or major reintervention in the row of the limbs with surgical reconstruction; 476 limbs (454 limbs) (**Table 5-6**). This is speculated to be due to death after reintervention. In **Table 5-6**, the objective for input of “revision for those excluding good bypass graft/EVT condition” is the limb registered in stenosis, occlusion, deterioration, anastomosis disruption (aneurysm), infection, and others of “bypass graft/EVT condition.” The total number of “the contralateral limb occlusive lesions” in **Table 5-7** is equal to that of “life prognosis” in **Table 5-1**. The information of the contralateral limb at death was registered in a dead case. The sum of the number of “treatment for contralateral limb” is less than that of “the contralateral limb occlusive lesions” because the objectives of “treatment for contralateral limb” excluded the limbs of (−) in “the contralateral limb occlusive lesions.” Since multiple registrations were possible, the sum of the number of “treatment for contralateral limb” was more than that of (−) in “the contralateral limb occlusive lesions.” When a patient died within 1 month, the information of “newly diagnosed malignant neoplasm” at death was registered in **Table 5-8**.

In addition to the above, there were some parts where the total number does not match in **Tables 5-1** to **5-8**. It might be because several items had multiple choices or missing values.

## 4. Conclusion

Vascular surgeons’ contribution in participating facilities registered a sufficient amount of detailed data during busy clinical practice, which has been gradually clarifying the current status of CLI treatment in Japan. Data on CLI in 2018 were clarified, after annual data in 2013–2017. The JCLIMB Committee is planning to continue publishing an annual report in the future. In 2017, the new concept, “chronic limb threatening ischemia,” was proposed instead of CLI,^13)^ and a new clinical guideline, the Global Vascular Guideline, was published instead of TASC in 2019.^14)^ The JCLIMB Committee ought to revise the survey items according to the Global Vascular Guideline, and a new registration form, which can be used in 2021, is being prepared.

The JCLIMB Committee expects that these study results will be fed back to clinical situations to help develop medical care for CLI and clinical studies using these data are ongoing. Facilities can participate in the JCLIMB at any time by contacting the JSVS secretariat for details.

## 5. Participant Facilities (84 Facilities in the Order of the Japanese Syllabary by Prefecture, Corporate Names are Omitted as a Rule)

Department of Vascular Surgery, Asahikawa Medical University Hospital

Department of Cardiovascular Surgery, National Hospital Organization Obihiro Hospital

Department of Cardiovascular Surgery, Sapporo Teishinkai Hospital

Department of Cardiovascular Surgery, Nayoro City General Hospital

Department of Cardiovascular Surgery, Hirosaki University Hospital

Department of Surgery, Iwate Prefectural Isawa Hospital

Department of Surgery, Iwate Prefectural Chubu Hospital

Department of Vascular Surgery, Morioka Yuai Hospital

Department of Surgery, JR Sendai Hospital

Department of Cardiovascular Surgery, Sendai City Hospital

Department of Transplantation, Reconstruction and Endoscopic Surgery, Tohoku University Hospital

Department of Cardiovascular Surgery, Saiseikai Yamagata Saisei Hospital

Department of Cardiovascular Surgery, Tokushukai Shonai Amarume Hospital

Department of Cardiovascular Surgery, Southern TOHOKU General Hospital

Department of Vascular and Endovascular Surgery, Ibaraki Prefectural Central Hospital

Department of Cardiac and Vascular Surgery, Dokkyo Medical University Nikko Medical Center

Department of Cardiac and Vascular Surgery, Dokkyo Medical University Hospital

Department of Vascular and Endovascular Surgery, International University of Health and Welfare

Department of Vascular Surgery, Saiseikai Kawaguchi General Hospital

Department of Vascular Surgery, Saitama Medical Center, Saitama Medical University

Department of Cardiovascular Surgery, Saitama Medical Center, Jichi Medical University

Department of Cardiovascular Surgery, Jichi Medical University

Department of Cardiovascular Surgery, Tokorozawa Meisei Hospital

Department of Surgery, Saitama City Hospital

Department of Cardiac and Vascular Surgery, National Defense Medical College Hospital

Department of Cardiovascular Surgery, Shimada General Hospital

Department of Cardiovascular Surgery, Chiba Cerebral and Cardiovascular Center

Department of Cardiovascular Surgery, Itabashi Chuo Medical Center

Department of Cardiovascular Surgery, IMS Tokyo Katsushika General Hospital

Department of Surgery, Tokyo Metropolitan Health and Medical Treatment Corporation, Okubo Hospital

Department of Cardiovascular Surgery, Kyorin University

Department of Surgery, Keio University School of Medicine

Department of Vascular Surgery, International University of Health and Welfare, Mita Hospital

Department of Vascular Surgery, Tokyo Medical and Dental University

Department of Cardiovascular Surgery, Tokyo Medical University Hachioji Medical Center

Department of Cardiovascular Surgery, Tokyo Medical University Hospital

Department of Vascular Surgery, The Jikei University Kashiwa Hospital

Department of Vascular Surgery, The Jikei University Hospital

Department of Cardiovascular Surgery, Tokyo Women’s Medical University Medical Center East

Department of Vascular Surgery, The University of Tokyo Hospital

Department of Cardiovascular Surgery, Tokyo Rinkai Hospital

Department of Vascular Surgery, Nihon University Itabashi Hospital

Department of Surgery, Shonan Kamakura General Hospital

Department of Vascular Surgery, Kawasaki Municipal Hospital

Department of Cardiovascular Surgery, St. Marianna University School of Medicine

Department of Surgery, Tomei Atsugi Hospital

Department of Cardiovascular Surgery, Yokosuka General Hospital Uwamachi

Department of Cardiovascular Surgery, National Hospital Organization, Kanazawa Medical Center

Department of Cardiovascular Surgery, Kanazawa University Hospital

Department of Cardiovascular Surgery, Shizuoka Red Cross Hospital

Department of Vascular Surgery, Aichi Medical University Hospital

Department of Vascular Surgery, Ichinomiya Municipal Hospital

Department of Vascular Surgery, Japanese Red Cross Nagoya Daiichi Hospital

Department of Vascular Surgery, Nagoya University Hospital

Department of Vascular Surgery, Osaka Rosai Hospital

Department of Vascular Surgery, Aijinkai Inoue Hospital

Department of Vascular Surgery, Nippon Life Hospital

Department of Vascular Surgery, Kansai Medical University Medical Center

Department of Cardiovascular Surgery, Toyonaka Municipal Hospital

Department of Cardiovascular Surgery, Tsukazaki Hospital

Department of Cardiovascular Surgery, Kobe University Hospital

Department of Thoracic and Cardiovascular Surgery, Wakayama Medical University Hospital

Department of Cardiovascular Surgery, Tottori Prefectural Kousei Hospital

Department of Cardiovascular Surgery, Tottori Prefectural Central Hospital

Department of Cardiovascular Surgery, Okayama University Hospital

Department of Cardiovascular Surgery, Kawasaki Medical School Hospital

Department of Peripheral Vascular Surgery, The Sakakibara Heart Institute of Okayama

Department of Cardiovascular and Respiratory Surgery, Hiroshima Prefectural Hospital

Department of Cardiovascular Surgery, National Hospital Organization, Higashihiroshima Medical Center

Department of Surgery, Hiroshima Red Cross Hospital & Atomic-bomb Survivors Hospital

Department of Cardiovascular Surgery, Hiroshima University Hospital

Department of Surgery, Saiseikai Yamaguchi General Hospital

Department of Surgery 1, Yamaguchi University Hospital

Department of Cardiovascular Surgery, Ehime Prefectural Central Hospital

Department of Cardiovascular Surgery, Ehime University Hospital

Department of Cardiovascular Surgery, Matsuyama Shimin Hospital

Department of Vascular Surgery, Matsuyama Red Cross Hospital

Department of Cardiovascular Surgery, Kochi Health Sciences Center

Department of Vascular Surgery, National Hospital Organization, Kyushu Medical Center

Department of Surgery and Science, Kyushu University Hospital

Department of Cardiovascular Surgery, Kurume University Hospital

Department of Vascular Surgery, Kokura Memorial Hospital

Department of Surgery, Saiseikai Fukuoka General Hospital

Department of Surgery, Saiseikai Yahata General Hospital

Department of Vascular Surgery, Fukuoka City Hospital

Department of Vascular Surgery, National Hospital Organization, Fukuokahigashi Medical Center

Department of Surgery, Saiseikai Karatsu Hospital

Department of Cardiovascular Surgery, Sasebo Chuo Hospital

Department of Vascular Surgery, Kumamoto Rehabilitation Hospital

Department of Cardiovascular Surgery, Oita Oka Hospital

## 6. JCLIMB Committee, NCD JCLIMB Analytical Team

### (1) JCLIMB Committee

Shinsuke Mii (Chairman), Kunihiro Shigematsu (Vice Chairman), Nobuyoshi Azuma, Atsuhisa Ishida, Yoshinori Inoue, Hisashi Uchida, Takao Ohki, Sosei Kuma, Koji Kurosawa, Akio Kodama, Hiroyoshi Komai, Kimihiro Komori, Takashi Shibuya, Shunya Shindo, Ikuo Sugimoto, Juno Deguchi, Katsuyuki Hoshina, Hideaki Maeda, Hirofumi Midorikawa, Terutoshi Yamaoka, Hiroya Yamashita, and Yasuhiro Yunoki, and Tetsuro Miyata (Observer)

### (2) NCD JCLIMB Analytical Team

Arata Takahashi and Hiroaki Miyata

## Additional Remarks

This report was authorized by the institutional review board of Saiseikai Yahata General Hospital (Authorization No. 163).

## Additional Note

The original Annual Report was published in Japanese Journal of Vascular Surgery Vol. 29 (2020) No. 6; however, errors in numerical data were detected after the publication. The errata were published in Vol. 30 (2021) Nos. 2 and 3 of the same journal. This translation reflects that correction.

## Notes

This is a translation of Jpn J Vasc Surg 2020; 29: 365–393.

## References

[1] Japanese Society for Vascular Surgery JCLIMB Committee, NCD JCLIMB Analytical Team. 2013 JAPAN Critical Limb Ischemia Database (JCLIMB) Annual Report. Ann Vasc Dis 2016; 9: 356-73.28018515

[2] Japanese Society for Vascular Surgery JCLIMB Committee, NCD JCLIMB Analytical Team. 2014 JAPAN Critical Limb Ischemia Database (JCLIMB) Annual Report. Ann Vasc Dis 2016; 9: 374-91.28018516

[3] Japanese Society for Vascular Surgery JCLIMB Committee, NCD JCLIMB Analytical Team. 2015 JAPAN Critical Limb Ischemia Database (JCLIMB) Annual Report. Ann Vasc Dis 2018; 11: 398-426. 30402196 10.3400/avd.ar.18-00048 PMC6200626

[4] Japanese Society for Vascular Surgery JCLIMB Committee, NCD JCLIMB Analytical Team. 2016 JAPAN Critical Limb Ischemia Database (JCLIMB) Annual Report. Ann Vasc Dis 2019; 12: 109-35. 30931073 10.3400/avd.ar.19-00012 PMC6434344

[5] Japanese Society for Vascular Surgery JCLIMB Committee, NCD JCLIMB Analytical Team. 2017 JAPAN Critical Limb Ischemia Database (JCLIMB) Annual Report. Ann Vasc Dis 2020; 13: 205-33. 32595803 10.3400/avd.ar.20-00018 PMC7315241

[6] Norgren L, Hiatt WR, Dormandy JA, et al. Inter-society consensus for the management of peripheral arterial disease (TASC II). J Vasc Surg 2007; 45 **Suppl S**: S5-67. 17223489 10.1016/j.jvs.2006.12.037

[7] Mills JL Sr, Conte MS, Armstrong DG, et al. The Society for Vascular Surgery Lower Extremity Threatened Limb Classification System: risk stratification based on wound, ischemia, and foot infection (WIfI). J Vasc Surg 2014; 59: 220-34, 2. 24126108 10.1016/j.jvs.2013.08.003

[8] Japanese Society of Nephrology. Clinical Practice Guidebook for Diagnosis and Treatment of Chronic Kidney Disease 2012. Tokyo: Tokyo Igakusya; 2012. (in Japanese)

[9] Taylor SM, Kalbaugh CA, Gray BH, et al. The LEGS score: a proposed grading system to direct treatment of chronic lower extremity ischemia. Ann Surg 2003; 237: 812-9; discussion, 818-9. 12796577 10.1097/01.SLA.0000071563.19208.50 PMC1514690

[10] Armstrong DG, Lavery LA, Harkless LB. Validation of a diabetic wound classification system. The contribution of depth, infection, and ischemia to risk of amputation. Diabetes Care 1998; 21: 855-9. 9589255 10.2337/diacare.21.5.855

[11] Yamada T, Ohta T, Ishibashi H, et al. Clinical reliability and utility of skin perfusion pressure measurement in ischemic limbs—comparison with other noninvasive diagnostic methods. J Vasc Surg 2008; 47: 318-23. 18241755 10.1016/j.jvs.2007.10.045

[12] Rutherford RB, Baker JD, Ernst C, et al. Recommended standards for reports dealing with lower extremity ischemia: revised version. J Vasc Surg 1997; 26: 517-38. 9308598 10.1016/s0741-5214(97)70045-4

[13] Aboyans V, Ricco JB, Bartelink MLEL, et al. 2017 ESC guideline on the diagnosis and treatment of peripheral arterial diseases, in collaboration with the European Society of Vascular Surgery (ESVS): document covering atherosclerotic disease of extracranial carotid and vertebral, mesenteric, renal, upper and lower extremity arteries. Eur Heart J 2018; 39: 763-816. 28886620 10.1093/eurheartj/ehx095

[14] Conte MS, Bradbury AW, Kolh P, et al. Global vascular guidelines on the management chronic limb-threatening ischemia. J Vasc Surg 2019; 69 **6S**: 3S-125S, e40. 31159978 10.1016/j.jvs.2019.02.016 PMC8365864

