# ERRATUM

**DOI:** 10.3400/avd.err.21-01000

**Published:** 2021-09-25

Vol .14 (2021) No. 2 pp. 202–230

2018 JAPAN Critical Limb Ischemia Database (JCLIMB) Annual Report

The Japanese Society for Vascular Surgery JCLIMB Committee

In the above secondary publication article, numerical errors in tables were found after the publication. The erratum of the original Japanese article was published in Japanese Journal of Vascular Surgery Vol. 30 (2021) No. 4. The corrected version of table is given on the next page.

p. 221, **Table 4-4**

Incorrect:

**Table:** Table 4-4 Treatment 4

a. Total																			
	Distal bypass																		
	Proximal anastomosis								Distal anastomosis		Distal anastomosis: sites of crural artery				Distal anastomosis: sites of foot artery				
	External iliac	Common femoral	Deep femoral	Superficial femoral	Proximal popliteal	Distal popliteal	Crural	Others	Crural	Foot	Tibioperoneal trunk	Posterior tibial	Anterior tibial	Peroneal	Posterior tibial	Anterior tibial	Peroneal	Dorsalis pedis	Plantar
Rutherford 4	0	14	2	8	6	4	2	1	17	19	2	12	1	2	7	2	1	9	0
Rutherford 5	5	46	6	36	25	89	5	7	79	137	6	34	31	10	22	20	5	78	15
Rutherford 6	0	6	3	6	4	18	3	1	13	28	0	7	4	2	8	2	0	16	3
Total	5	66	11	50	35	111	10	9	109	184	8	53	36	14	37	24	6	103	18
b. ASO																			
	Distal bypass																		
	Proximal anastomosis								Distal anastomosis		Distal anastomosis: sites of crural artery				Distal anastomosis: sites of foot artery				
	External iliac	Common femoral	Deep femoral	Superficial femoral	Proximal popliteal	Distal popliteal	Crural	Others	Crural	Foot	Tibioperoneal trunk	Posterior tibial	Anterior tibial	Peroneal	Posterior tibial	Anterior tibial	Peroneal	Dorsalis pedis	Plantar
Rutherford 4	0	14	2	8	6	3	2	1	17	18	2	12	1	2	6	2	1	9	0
Rutherford 5	5	44	6	35	25	82	5	7	75	131	5	32	31	9	20	20	5	77	12
Rutherford 6	0	6	3	6	4	18	2	1	13	27	0	7	4	2	7	2	0	16	3
Total	5	64	11	49	35	103	9	9	105	176	7	51	36	13	33	24	6	102	15

Correct:

**Table:** Table 4-4 Treatment 4

a. Total																			
	Distal bypass																		
	Proximal anastomosis								Distal anastomosis		Distal anastomosis: sites of crural artery				Distal anastomosis: sites of foot artery				
	External iliac	Common femoral	Deep femoral	Superficial femoral	Proximal popliteal	Distal popliteal	Crural	Others	Crural	Foot	Tibioperoneal trunk	Posterior tibial	Anterior tibial	Peroneal	Posterior tibial	Anterior tibial	Peroneal	Dorsalis pedis	Plantar
Rutherford 4	0	13	2	7	2	10	4	2	21	21	0	13	8	0	5	5	2	8	2
Rutherford 5	4	46	5	30	24	77	9	2	71	124	3	40	20	10	30	9	3	71	12
Rutherford 6	1	11	0	9	8	22	0	0	19	33	0	10	6	3	11	2	0	16	6
Total	5	70	7	46	34	109	13	4	111	178	3	63	34	13	46	16	5	95	20
b. ASO																			
	Distal bypass																		
	Proximal anastomosis								Distal anastomosis		Distal anastomosis: sites of crural artery				Distal anastomosis: sites of foot artery				
	External iliac	Common femoral	Deep femoral	Superficial femoral	Proximal popliteal	Distal popliteal	Crural	Others	Crural	Foot	Tibioperoneal trunk	Posterior tibial	Anterior tibial	Peroneal	Posterior tibial	Anterior tibial	Peroneal	Dorsalis pedis	Plantar
Rutherford 4	0	11	2	7	1	9	4	1	19	18	0	11	8	0	4	5	1	8	1
Rutherford 5	4	45	5	29	24	71	9	2	67	120	3	38	20	8	27	9	3	70	12
Rutherford 6	1	11	0	9	8	18	0	0	19	29	0	10	6	3	10	2	0	14	4
Total	5	67	7	45	33	98	13	3	105	167	3	59	34	11	41	16	4	92	17

